# Diversification and Demography of the Oriental Garden Lizard (*Calotes versicolor*) on Hainan Island and the Adjacent Mainland

**DOI:** 10.1371/journal.pone.0064754

**Published:** 2013-06-26

**Authors:** Yong Huang, Xianguang Guo, Simon Y. W. Ho, Haitao Shi, Jiatang Li, Jun Li, Bo Cai, Yuezhao Wang

**Affiliations:** 1 Department of Herpetology, Chengdu Institute of Biology, Chinese Academy of Sciences, Chengdu Sichuan, P.R. China; 2 Guangxi Botanical Garden of Medicinal Plants, Nanning Guangxi, P.R. China; 3 School of Biological Sciences, University of Sydney, New South Wales, Australia; 4 College of Life Sciences, Hainan Normal University, Haikou Hainan, P.R. China; Onderstepoort Veterinary Institute, South Africa

## Abstract

The Oriental garden lizard (*Calotes versicolor*) is one of the few non-gekkonid lizards that are geographically widespread in the tropics. We investigated its population dynamics on Hainan Island and the adjacent mainland of China and Vietnam, focusing on the impact of cyclic upheaval and submergence of land bridges during the Pleistocene. Our Bayesian phylogenetic analysis reveals two mitochondrial lineages, A and B, which are estimated to have coalesced about 0.26 million years ago (95% credibility interval: 0.05–0.61 million years ago). Lineage A contains individuals mainly from central and southern Wuzhi Mountain on Hainan Island, whereas lineage B mainly comprises individuals from other sites on the island plus the adjacent mainland. The estimated coalescence times within lineages A (0.05 million years ago) and B (0.13 million years ago) fall within a period of cyclical land-bridge formation and disappearance in the Pleistocene. A spatial analysis of molecular variance identified two distinct population groupings: I, primarily containing lineage A, and II, mainly consisting of lineage B. However, haplotypes from lineages A and B occur sympatrically, suggesting that gene flow is ongoing. Neither Wuzhi Mountain nor Qiongzhou Strait and Gulf of Tonkin act as barriers to gene flow among *C. versicolor* populations. Analyses of the data using mismatch distributions and extended Bayesian skyline plots provide evidence of a relatively stable population size through time for Group I, and moderate population expansions and contractions during the end of the Pleistocene for Group II. We conclude that the phylogeographical patterns of *C. versicolor* are the combined product of Pleistocene sea-level oscillations and nonphysical barriers to gene flow.

## Introduction

The geographical distributions and genetic structure of organisms in the present have been shaped by factors such as climatic oscillations and geological events during the Pleistocene [1]–[3]. A cycle of glacial and interglacial climatic conditions dominated this epoch, causing sea-level changes with amplitudes of up to 120–140 m [4]. These led to the cyclic upheaval and submergence of land bridges between landmasses, repeatedly dissecting and rejoining populations and producing intermittent range contractions and expansions in many species [2]. Island populations that have been isolated from their mainland counterparts can exhibit genetic divergence, local adaptation, and signs of incipient speciation. On the other hand, transient land bridges may facilitate gene flow in both directions [1]. Taking these into account, one may reasonably assume that climate-mediated changes in sea level are likely to have contributed to genetic variation and divergence among populations and species.

Significant geological events, such as the formation of mountain ranges, are often treated as a source of long-term biogeographical barriers when inferring present or past patterns of gene flow [5]. In some cases, however, distinct phylogeographical patterns can arise without obvious geographical barriers (e.g ecological divergence, geographical distance, competition) to gene flow among populations [1], [6]. Ecological interactions of organisms with their environment play an important role in population divergence and speciation [7]. The use of such data in ecological niche modeling [8] has provided insights into speciation and diversification in relation to environmental factors or niche divergences. It is becoming increasingly common to incorporate ecological data into studies of mechanisms driving diversification and demography [9]. In contrast with the large body of examples of phylogeographical structuring caused by physical barriers, there is a poor understanding of nonphysical barriers to gene flow [10].

Island systems can provide compelling environments for evolutionary studies of species [11], especially if they have been connected to other landmasses in the past. One such example is Hainan Island, located in the transitional zone between tropical and temperate zones in the South China Sea. Part of the Sunda Shelf, the island is separated from the Chinese mainland to the north by Qiongzhou Strait, with a minimum gap of 19 km of open sea, and from the Vietnamese mainland to the west by the Gulf of Tonkin. Hainan Island was connected to mainland China until the appearance of Qiongzhou Strait, which formed as a result of volcanism and rising sea levels about 2–2.5 million years ago (Ma) [12], [13]. Subsequently, sea-level changes during the Pleistocene caused the island to be separated repeatedly from mainland China. Land bridges formed three times in the Middle Pleistocene: 0.6–0.8 Ma, 0.42–0.48 Ma, and 0.13–0.3 Ma [14]. Hainan Island was also connected to the mainland during the last glacial maximum (0.015–0.025 Ma; [15]) but has been separated since around 0.0071–0.01 Ma [13]. Although the separation time is recent, investigations of geographical patterns and genetic structure among populations on both sides of the strait can increase our understanding of any ecological processes that have had a phylogeographical influence [16].

**Figure 1:**
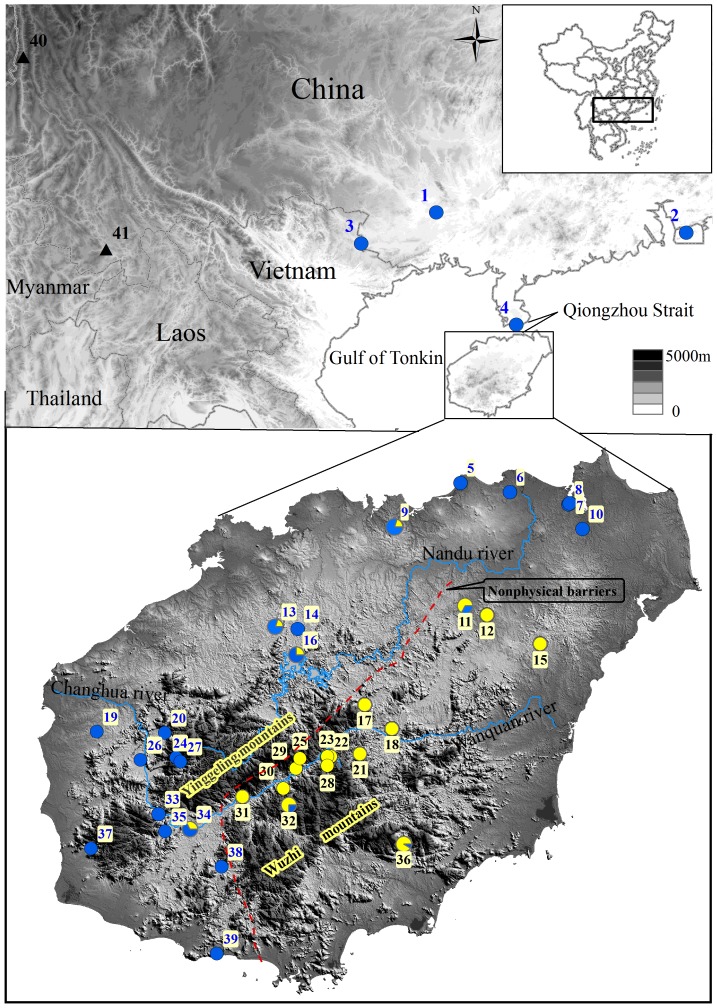
Sampling localities of *Calotes versicolor* on Hainan Island and the mainland. Samples are numbered following Table 1. Yellow and blue circles represent lineage A haplotypes and lineage B haplotypes, respectively. The sampled *C. versicolor*-like population of Yunnan is indicated by the black triangle. Populations with mixed haplotypes are represented by pie charts indicating the proportion of each haplotype. The approximate position of the non-physical barrier is shown.

Hainan Island is characterized by two zoogeographical regions, the mountain provinces and plain provinces, which are defined by altitude, climate, vegetation, and distribution of terrestrial vertebrates [17]. The topography of Hainan Island is diverse, with Wuzhi and Yinggeling Mountains approaching elevations of 1800 m, rising steeply from the central and southern regions and giving way to a broad plain in the north. The three largest rivers on the island, the Nandu, Changhua, and Wanquan, originate from the central mountainous area and flow outwards into the South China Sea (Figure 1).

There are 28 lizard species belonging to eight families on Hainan Island. Among these is the Oriental garden lizard, *Calotes versicolor*, an insectivorous agamid that is distributed across Asia. It ranges from Oman, across southern and South-East Asia to Indo-China, the Maldives, Réunion, Mauritius, and Seychelles, and was recently introduced to Florida in the USA [18]–[22]. This extensive range makes *C. versicolor* one of the most widespread non-gekkonid lizards in the world [23]. Zug *et al.* [24] used mitochondrial DNA and morphological characters to investigate the systematics of this species in Myanmar. They found evidence of a species complex with deep intraspecific genetic differentiation, in which at least two distinct evolutionary lineages were identified, albeit without a differentiated morphotype for each lineage.

*Calotes versicolor* is common on Hainan Island and the adjacent mainland (i.e., Vietnam and the Chinese provinces of Guangdong and Guangxi). This provides an opportunity for assessing the phylogeographical effects of past land bridges and contemporary geographical separation. In the present study, we sampled individuals across most of the geographical range of *C. versicolor* on Hainan Island, and conducted a range of phylogenetic and population-genetic analyses of their mitochondrial DNA. The objectives of our study are: (1) to estimate the impact of Pleistocene climatic variations on the genetic structure of the species; (2) to test whether Qiongzhou Strait and Wuzhi Mountain impede gene flow between populations; and (3) to investigate past population dynamics of *C. versicolor*, especially on Hainan Island.

## Materials and Methods

### Ethics statement

According to “Law of People's Republic of China on the Protection of Wildlife” and “Regulations for the Implementation of the People's Republic of China on the Protection of terrestrial Wildlife” (State Council Decree [1992] No. 13), this study did not require any ethical or institutional approvals since *Calotes versicolor* is not endangered or protected by any law. All samples obtained in these locations in field were not required any specific permissions because all sampled locations are not privately owned or protected. Collections of tissue samples were carried out in strict accordance with “Regulation for the Collection of Genetic Resources (HJ 628–2011)”, and all practical efforts were made to ameliorate specimens suffering throughout this study. This study was approved by the Animal Ethics Committee of Chengdu Institute of Biology, Chinese Academy of Sciences, and animal experiments were carried out in line with the institutional guidelines.

### Sample collection and DNA sequencing

We sampled 212 individuals of *C. versicolor* from 39 sites between 2007 and 2009. These included 191 individuals from 35 sites across Hainan Island, covering the entire range of the species on the island to allow us to characterize its lineage differentiation, biogeographical patterns, and historical demography. In order to infer the expected impact of sea-level fluctuations at lower latitudes on the species, we also sampled 21 individuals from 4 sites on the Chinese mainland (Guangdong, Guangxi, and Hong Kong) and Vietnam (Figure 1 and Table 1). Additionally, we sampled three *C. versicolor*-like individuals from two sites in Yunnan, China. Specimens were euthanized in the field, with liver tissue kept in 95% ethanol using cryo tubes. All voucher specimens were deposited in the herpetological collections of the Chengdu Institute of Biology, Chinese Academy of Sciences. We obtained sequences from GenBank for a number of outgroup sequences (*Iguana iguana*, *Chamaeleo africanus*, *Chamaeleo dilepis*, *Leiolepis belliana*, *Physignathus cocincinus*, *Pogona vitticepes*, *Hydrosaurus amboinensis*, *Agama agama*, *Agama bibronii*, *Laudakia caucasia*, *Laudakia microlepis*, *Acanthosaura armata*, *Calotes emma*, *Calotes liolepis*, *Calotes irawadi*, *Draco blanfordii*; Table S1).

**Table 1:** Sample localities, sample sizes, number of haplotypes (*N*), haplotype diversity (*h*), and nucleotide diversity (*π*) for *Calotes versicolor*.

Population	Locality	Sample size	Longitude	Latitude	Genetic diversity		
					*N*	*h*	*π*
1	Nanning	3	108.37	22.86	2	0.667	0.0003
2	Hongkong	1	114.11	22.40	1	0.000	0.0000
3	Vietnam	8	106.65	22.15	4	0.643	0.0011
4	Haian	9	110.21	20.28	5	0.806	0.0009
	**Mainland**	21	–	–	12	0.914	0.0026
5	Changliu	1	110.16	20.03	1	0.000	0.0000
6	Haikou	5	110.34	20.00	2	0.400	0.0002
7	Yanfeng2	2	110.56	19.96	2	1.000	0.0045
8	Yanfeng1	1	110.55	19.95	1	0.000	0.0000
9	Fushan	5	109.92	19.87	5	1.000	0.0093
10	Wenchang	1	110.60	19.86	1	0.000	0.0000
11	Tunchang	3	110.18	19.58	3	1.000	0.0118
12	Fuwen	2	110.26	19.55	1	0.000	0.0000
13	Nada2	16	109.48	19.51	10	0.917	0.0100
14	Nada1	1	109.56	19.50	1	0.000	0.0000
15	Huangzhu	4	110.45	19.44	3	0.833	0.0014
16	Nanfeng	7	109.56	19.40	4	0.810	0.0096
17	Limushan	5	109.81	19.22	3	0.700	0.0007
18	Wanling	6	109.91	19.13	4	0.800	0.0006
19	Datian	7	108.83	19.12	5	0.857	0.0046
20	Wanting	5	109.08	19.12	3	0.700	0.0053
21	Jiachai	2	109.79	19.04	2	1.000	0.0015
22	Fanxiang	7	109.67	19.03	6	0.952	0.0017
23	Hongmao	2	109.68	19.03	2	1.000	0.0026
24	Bawangling	6	109.12	19.03	5	0.933	0.0034
25	Shiyun	1	109.57	19.02	1	0.000	0.0000
26	Donghe	6	108.99	19.02	4	0.867	0.0052
27	Sanpai	14	109.14	19.01	4	0.626	0.0047
28	Hela	4	109.67	19.00	2	0.500	0.0008
29	Chonggongbao	6	109.56	18.99	4	0.800	0.0011
30	Maoyang	1	109.51	18.91	1	0.000	0.0000
31	Fanyang	7	109.36	18.88	4	0.857	0.0012
32	Hongshan	7	109.53	18.86	4	0.810	0.0057
33	Jiangbian	2	109.06	18.82	2	1.000	0.0026
34	Yongming	4	109.17	18.77	3	0.833	0.0097
35	Zhiwei	8	109.08	18.76	6	0.929	0.0026
36	Lingshui	18	109.95	18.71	11	0.889	0.0036
37	Jianfeng	7	108.81	18.70	4	0.810	0.0034
38	Zhizhong	7	109.29	18.63	6	0.952	0.0012
39	Tianya	11	109.27	18.31	4	0.746	0.0007
	**Island**	191	–	–	105	0.990	0.0109
	**Total**	212	–	–	117	0.991	0.0108
40	Gaoligong	2	98.90	26.42	2	1.000	0.0026
41	Xishuangbanna	1	100.80	22.01	1	0.000	0.0000

Total genomic DNA was extracted from tissue samples following a universal protocol of DNA extraction [25]. A region of the mitochondrial genome spanning *tRNA_Trp_*, the *ND2* gene, and the *COI* gene was targeted. All samples were amplified and sequenced with our designed primers, L3705 (5′-ATT AGG GTC TGC TAC ACA AGC AGT TGG-3′) and H5162 (5′-GGT TGA RAG TAR TCA TCG AGT TAA GAA CGAC-3′), as well as L5037 (5′-GAG TAG ACC CAG GAA CCR AAG TTC-3′) in combination with H6448 (5′-GTA TAC CGG CTA ATC CAA GCA TGT G-3′). Standard polymerase chain reactions (PCR) were performed in 50 µL reactions, including approximately 100 ng of template DNA (1 ng/µL), 2 µL of each primer (each 1 µmol/µL), 5 µL of 10×*Ex*-*Taq* buffer (Mg^2+^ Plus), 4 µL dNTPs (each 2.5 mmol/L), 0.3 µL of *Ex*-*Taq* DNA polymerase (5 U/µL), and sterile distilled water. PCR was conducted with the following conditions: an initial denaturing step at 95°C for 4 min; 35 cycles of denaturing at 94°C for 35 s, annealing at 65°C for 45 s, and extending at 72°C for 90 s; and a final extending step of 72°C for 8 min. PCR products were electrophoresed in 0.8% agarose gels, visualized with ethidium bromide. These products were purified and sequenced directly by Invitrogen Trading (Shanghai) Co., Ltd, using the corresponding PCR primers. Where products could not be sequenced by the primers above, we used L3705 in combination with L4375 (5′-CAC CTA CAT CAY CCT AAC-3′). All individuals were sequenced in both directions. All new sequences were deposited in GenBank with accession numbers KC875609 to KC875820.

### Phylogenetic and molecular dating analyses

Nucleotide sequences were checked and aligned by eye. No premature stop codons were observed in the *ND2* and *COI* regions, suggesting that the sequences were not nuclear copies of mitochondrial sequences. We used DnaSP v5.0 [26] to calculate the number of haplotypes and haplotype diversity (*h*) and to estimate nucleotide diversities (*π*) [27] for the total sample and for each population (Table 1).

To estimate the phylogeny and divergence times for the mitochondrial haplotypes, we conducted a Bayesian analysis using the program BEAST v1.5.4 [28]. Using a relaxed molecular clock enabled us to estimate divergence times in the presence of rate heterogeneity among lineages [29]. Estimating molecular divergence times requires *a priori* assumptions about the age of one or more clades to calibrate the substitution rate. This is most commonly achieved by using fossil calibrations. In the absence of fossil calibrations for *C. versicolor*, we expanded the data set to include various agamid taxa from GenBank (Table S1). We also included sequences from the *C. versicolor* group from the Central Dry Zone of Myanmar [24]. We partitioned the data set by gene and by codon position, producing seven partitions. The most appropriate evolutionary model was selected for each partition using the hierarchical likelihood-ratio test implemented in Modeltest v3.7 [30].

**Figure 2:**
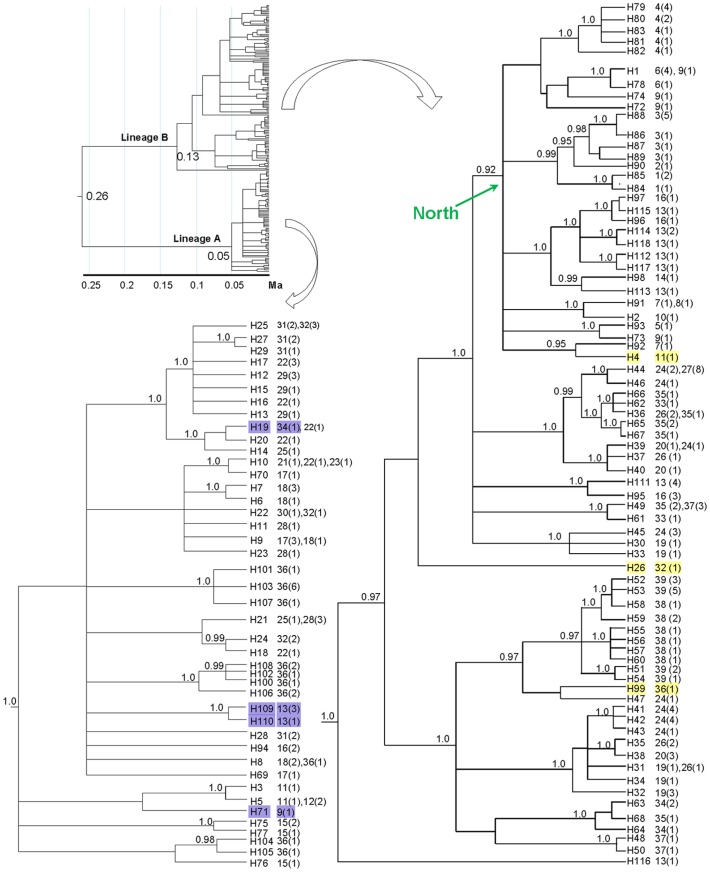
Relationships of all *Calotes versicolor* individuals inferred from mtDNA sequence data using Bayesian phylogenetic analysis. The 50% majority-rule consensus tree shows the 118 unique haplotypes of *C. versicolor*. Numbers outside parentheses represent population codes following Table 1, whereas values within parentheses indicate the number of sampled individuals exhibiting that haplotype. Branch lengths are in units of time. Posterior probabilities above 0.9 are shown next to nodes. Haplotypes with yellow rectangles in lineage B represent the individuals mainly collected from Wuzhi Mountain, whereas haplotypes with blue rectangles in lineage A represent the individuals collected from the remaining part of Hainan Island and the adjacent mainland.

We estimated the phylogeny and divergence times of 28 outgroup taxa and two randomly sampled haplotypes that spanned the root of the *C. versicolor* tree (Figure 2). The use of deep fossil calibrations can lead to overestimates of recent divergence times and molecular rates, probably owing to an excess of deleterious mutations at the population level [31], [32]. Nonsynonymous sites show stronger time-dependent pattern of rates than synonymous sites, resulting in overestimates of molecular rates on short timescales [33], [34]. Therefore, we used a two-step approach similar to that employed by Pepper *et al.* [35]. We began by analysing the third codon sites of mitochondrial protein-coding sequences, which reduces the impact of purifying selection on estimates of evolutionary timescales [36]. Our first step was to use a calibration for the divergence of *Laudakia microlepis* and *L. caucasia*. This split was constrained to be 8–10 Ma, assuming vicariance based on mountain uplifts caused by the collision of the Indian and Arabian plates [37]. A lognormal prior distribution (mean = 0, standard deviation = 0.354, offset = 8) was chosen to reflect the uncertainty in the fossil calibration age. Posterior distributions of parameters were estimated using Markov chain Monte Carlo (MCMC) sampling. Samples were drawn every 1,000 steps over a total of 5×10^7^ MCMC steps. We used a Yule tree prior with an uncorrelated lognormal relaxed clock [29].

In the second step of our analysis, we ran a partitioned BEAST analysis including all 212 sequences of *C. versicolor*, using the age estimate for the most recent common ancestor of the two *C. versicolor* lineages from the first step (Figure S1). This calibration was implemented as a lognormal prior, with mean = 0.57, log (stdev) = 0.72, and offset = 0.03. A constant-size coalescent prior was used for the tree. The optimal substitution model was selected for each data partition. Samples from the posterior were drawn every 1,000 steps over a total of 2×10^7^ MCMC steps. We conducted four replicates of each BEAST analysis and compared their results to check for MCMC convergence and acceptable mixing. The results were found to be satisfactory and samples from the multiple runs were combined. We regarded posterior probabilities ≥0.95 as being indicative of strongly supported nodes [38].

### Population genetic analyses

To characterize population structure and to define groups of populations using genetic criteria, we conducted spatial analysis of molecular variance with the program SAMOVA 1.0 [39]. The analysis was run for values of *K* from 2 to 20 and the significance of fixation indices was tested using 1,000 random permutations. We chose the optimal number of groups based on two criteria. First, from a plot of *F_CT_* as a function of *K*, we determined the value of *K* necessary for *F_CT_* to reach a plateau. Second, we excluded the configurations of *K* that had one or more single-population groups, because this indicated that the group structure was disappearing [40], [41].

To test further for geographical genetic structure, analyses of molecular variance (AMOVA) with 10,000 permutations were assessed in Arlequin v3.1 [42], according to the degree of differentiation between regions (*F_CT_*), between populations within regions (*F_SC_*), and between all populations (*F_ST_*). We examined several hypotheses, including: (i) no overall regional structure; (ii) structure between Hainan Island and its adjacent mainland (the “Qiongzhou Strait isolation” hypothesis); (iii) structure between the mountain and plain provinces on Hainan Island (the “two zoogeographical regions” hypothesis); and (iv) structure caused by the Changhua-Wanquan River (Figure 1) presenting a barrier between the northwestern and southwestern groups. Separate AMOVA analyses were performed for the full data set and for the different lineages identified in the phylogenetic analysis, using the substitution model selected by the hierarchical likelihood-ratio test to calculate the distance matrix. Population groupings for each analysis are detailed in Table 2, with locations shown in Figure 1.

**Table 2:** Hierarchical analysis of AMOVA of *Calotes versicolor*.

Hypothesized structure	*F_ST_*	*F_SC_*	*F_CT_*	Among groups (%)	Among populations within groups (%)	Within populations (%)
All lineages						
No structure	0.66***	–	–	–	65.53	34.47
2 Groups: [Group I][Group II]	0.77***	0.33***	0.66***	65.77	11.63	22.60
2 Groups: [Group I][Group II]#	0.75***	0.29***	0.65***	64.95	10.20	24.85
2 Groups: [Hainan Island][Mainland]	0.71***	0.64***	0.19*	19.22	51.70	29.08
2 Groups: [Northwestern Changhua-Wanquan River][Southeastern Changhua-Wanquan River]#	0.62***	0.63***	−0.02	−2.52	65.24	37.28
2 Groups: [Mountain province in the central Hainan Island][Plain province along the coast Hainan Island]#	0.64***	0.62***	0.06	5.72	58.78	35.50
2 Groups: [Mountain province in the Hainan sub-zoogeographical region of South China][Plain province in the Hainan sub-zoogeographical region of South China]	0.66***	0.62***	0.10	10.21	56.24	33.55
3 groups: [Mountain province in the central Hainan Island][Plain province along the coast Hainan Island][Mainland]	0.68***	0.63***	0.12*	12.43	55.30	32.27
4 groups: [Mountain province in the central Hainan Island][Plain province along the coast Hainan Island][Chinese Mainland][Vietnam]	0.67***	0.64***	0.10	9.53	57.67	32.80
4 groups: [Mountain province in the Hainan sub-zoogeographical region of South China][Plain province in the Hainan sub-zoogeographical region of South China][Nanning][Vietnam]	0.67***	0.64***	0.10	10.15	57.23	32.62

The geographical division of the Groups I and II correspond to the ranges as shown in Figure 1.

# Excluding mainland populations, living Hainan Island populations.

* *P*<0.05;

*** *P*<0.001.

To test for associations between genetic and geographical distance among populations or within lineages, isolation-by-distance analyses [43] were performed using IBDWS v3.16 [44]. Significance was evaluated using 10,000 random permutations. Considering the two-dimensional habitats of *C. versicolor* across the sampled region, pairwise values of *F_ST_*/(1−*F_ST_*) were plotted against the logarithm of geographical distance (two-dimensional stepping-stone model) [45]. *F_ST_* values were calculated with Arlequin 3.1 using the TrN model of evolution selected by the hierarchical likelihood-ratio test. Reduced major axis regression was used to estimate the slope and intercept of the isolation-by-distance association. To include geographical structuring among populations, a third matrix (indicator matrix) was included so that populations were grouped by regions based on the results of AMOVA and SAMOVA tests and the “Qiongzhou Strait isolation” hypothesis. Partial Mantel tests took these groups into account while determining the significance of genetic and geographical distance relationships.

### Demographic analyses

To investigate past changes in the population size and range of *C. versicolor*, mismatch distributions were calculated for each group in Arlequin 3.1. To compare the observed data with the expected data under the sudden- and spatial-expansion models, we conducted goodness-of-fit tests based on the sum of squared deviations and Harpending's raggedness index [46] using 2,000 parametric bootstrap replicates. We also calculated Tajima's *D* [47] and Fu's *Fs* [48] statistics as an additional assessment of possible population expansion, with 10,000 coalescent simulations. Smooth and unimodal mismatch distributions with significantly negative values of Fu's *Fs* and Tajima's *D* suggest a recent population expansion, whereas multimodal mismatch distributions suggest that populations were recently influenced by migration, are subdivided, and/or have undergone historical contraction [49], [50]. The approximate expansion time of each group was estimated with the equation *t* = *τ*/2*u* [51], in which t is measured in generations, *τ* is measured in mutations per sequence, and *u* is the mutation rate per sequence per generation. The *u* value was calculated by formula *u* = 2*μk*, where *μ* is the mutation rate per nucleotide per generation and *k* is the number of nucleotides (2,663 bp). Finally, the approximate expansion time measured in years was estimated by multiplying *t* by the generation time of *C. versicolor* (one generation per year).

In order to examine the shape of population growth over time, we analysed the data using the extended Bayesian skyline plot [52] in BEAST. We assumed a strict molecular clock with a mean substitution rate (5.87% per site per million years) estimated from the secondary BEAST analysis described above. The two genetic groups were analysed separately. Samples were drawn from the posterior distribution every 1,000 MCMC steps, over a total of 5×10^7^ steps. The initial 10% of steps was discarded as burn-in. We obtained consistent demographic inferences across three replicates of the analysis.

### Ecological niche modeling

We firstly used niche models to test the ecological divergence between the different groups using Maxent version 3.3.1 [53], implementing 19 climatic layers downloaded from the WorldClim database (http://www.worldclim.org/) at 30 s resolution [54] as environment layers (Table S2). We used the default settings, with 75% of species records for training and 25% for testing the model, and with 100 replicate bootstrap samples and 5000 iterations to have adequate time for convergence. To determine whether species pairs have significantly different niches, we conducted a niche identity test using ENMtools following the methods described in [55]. This test compares niche models generated from the actual data for each species to the null models generated from the pooled sample of all actual occurrence localities. We calculated Schoener's *D* [55], and Hellinger's-based *I* [56] to measure niche identity. Schoener's *D* measures the similarity of two habitat niches, comparable to a percentage overlap [57]. Hellinger's-based *I* measures the probability distributions of two ecological niche models [58], comparable to other niche overlap metrics [59]. Both measures range from 0 (no niche overlap) to 1 (identical niches). Bootstrapping with 100 replicates was conducted using the pooled localities for two groups. Subsequently, a point-based method was used to assess whether ecological niche space of these genetic groups was differentiated. We extracted pixel values for each of the 19 bioclimatic variables at each point locality. Principal components analysis (PCA) was used to convert the original 19 climatic variables to principal components that account for most of the variability. To determine whether the ecological niches of the groups were statistically significant, a multivariate analysis of variance (MANOVA) was then performed. The groups were the fixed factor and PCA scores were the dependent variables. All of these significance analyses were performed with SPSS 19.0 [60].

## Results

### DNA sequence variation and genetic diversity

For each of the 212 individuals of *C. versicolor* sampled in our study, we sequenced 2663 bp of mitochondrial DNA, spanning *tRNA_Trp_*, *ND2*, and *COI*. Among the 118 haplotypes identified, only one (0.8%; haplotype 10) was shared by individuals from three sites (localities 21, 22, and 23). Two haplotypes (11.9%) were shared by individuals from two localities, whereas the remaining haplotypes (87.3%) were restricted to single sampling localities. The sequence alignment contained 239 variable nucleotide sites, of which 160 were parsimony-informative.

Nucleotide diversity (*π*) and haplotype diversity (*h*) within populations are summarized in Table 1. Values of *π* and *h* varied considerably among populations. The number of haplotypes (*N*) within each population ranged from 1 to 11, with the greatest number of haplotypes in the Lingshui population. Values of *π* ranged from 0 to 0.0012, whereas *h* ranged from 0 to 1.0000. Among all samples (excluding Gaoligong and Xishuangbanna populations), mean values of *h* and π were 0.991 and 0.0108, respectively, indicating a high haplotype diversity but relatively low nucleotide diversity (Table 1). These suggest that most populations of this species harbored a high frequency of private haplotypes (Table 1).

### Phylogeny and coalescence times

Bayesian phylogenetic analysis of the mitochondrial sequence data yielded strong support for monophyly of *C. versicolor*, with a posterior probability (PP) of 1.0 (Figure 2). The sampled haplotypes yielded two major lineages, A and B. Lineage A contained individuals mainly from central and southern Wuzhi Mountain on Hainan Island (PP = 1.0), whereas lineage B mainly comprised individuals from other sites on Hainan Island and the adjacent mainland (PP = 1.0; Figure 2). None of the haplotypes is geographically widespread. Most of the shared haplotypes occur within the same population or between closely related populations. However, the haplotypes from some populations fall into more than one regional phylogeographical group. For example, several haplotypes from Wuzhi Mountain (populations 11, 32, and 36, identified by yellow rectangles in Figure 2) are found in both lineages A and B, as are several haplotypes from the remaining part of Hainan Island (populations 9, 13, 16, and 34, identified by blue rectangles in Figure 2).

The coalescence time of all of the sampled individuals was estimated at 0.26 million years ago (95% credibility interval, 0.06–0.61 Ma), during a period of marked glacial cycling in the Pleistocene. The estimated coalescence times of lineages A and B are 0.05 Ma (95% CI: 0.01–0.13 Ma) and 0.13 Ma (95% CI: 0.02–0.31 Ma), respectively.

### Population genetic analyses

In the SAMOVA analysis, *F_CT_* values did not have the highest differentiation among groups when *K* = 2; however, one or more groups contained a single population when *K*≥3. Therefore, we retained the configuration of *K* = 2. For the AMOVA analysis, based on several hypotheses (see the Methods), the maximal *F_CT_* was achieved with two groups (*F_CT_* = 0.66, *P*<0.001, Table 2), showing the same pattern of grouping as that obtained in the SAMOVA tests. Populations within groups and within populations accounted for 11.63% and 22.60%, respectively, of the genetic variation. One group (Group I) included populations 11–12, 15, 17–18, 21–23, 25, 28–32, and 36, and the other group (Group II) included populations 1–10, 13–14, 16, 19–20, 24, 26–27, 33–35, and 37–39. The geographical division of the two groups corresponded to the ranges shown in Figure 1. The two groups, which excluded the mainland populations, also revealed significant genetic structure (*F_CT_* = 0.65, *P*<0.001; Table 2).

We found significant genetic structure for all individuals (*F_ST_* = 0.66, *P*<0.001; Table 2). There was evidence of significant structure between Hainan Island groups and the adjacent mainland groups (*F_CT_* = 0.20, *P*<0.05; Table 2), in accordance with the “Qiongzhou Strait isolation” hypothesis. We also found structure between groups among mountain and plain provinces in the Hainan Island and mainland groups (*F_CT_* = 0.12, *P*<0.05; Table 2). However, there was no significant structure between northwestern and southeastern groups (*F_CT_* = −0.25, *P*>0.05; Table 2), despite the barrier presented by the Changhua-Wanquan River. Likewise, genetic variation was not significant between the mountain and plain provinces in the Hainan sub-zoogeographical region, as would be expected from the “two zoogeographical regions” hypothesis.

We found a significant relationship between genetic and geographical distances for all populations. The reduced major axis regression had a slope of 3.48 (SE = 0.12) and an intercept of −6.78 (SE = 0.25), with an explanatory variation of 0.10. The Mantel test for matrix correlation of log-genetic and log-geographical distances was significant (z-value = 317.53, r = 0.31, one-sided *P*<0.0001). While controlling for indicator matrix based on the two most parsimonious geographical groups I and II inferred from both AMOVA and SAMOVA tests, the partial Mantel test was significant (r = 0.30, one-sided *P*<0.0001). To test the “Qiongzhou Strait isolation” hypothesis, partial correlation of genetic distance and geographical distance was r = 0.28 (one-sided *P*<0.0001) while controlling for indicator matrix. In contrast, when geographical distance was controlled, partial correlation was r = −0.05 (one-sided *P* = 0.76).

### Past population dynamics

**Figure 3:**
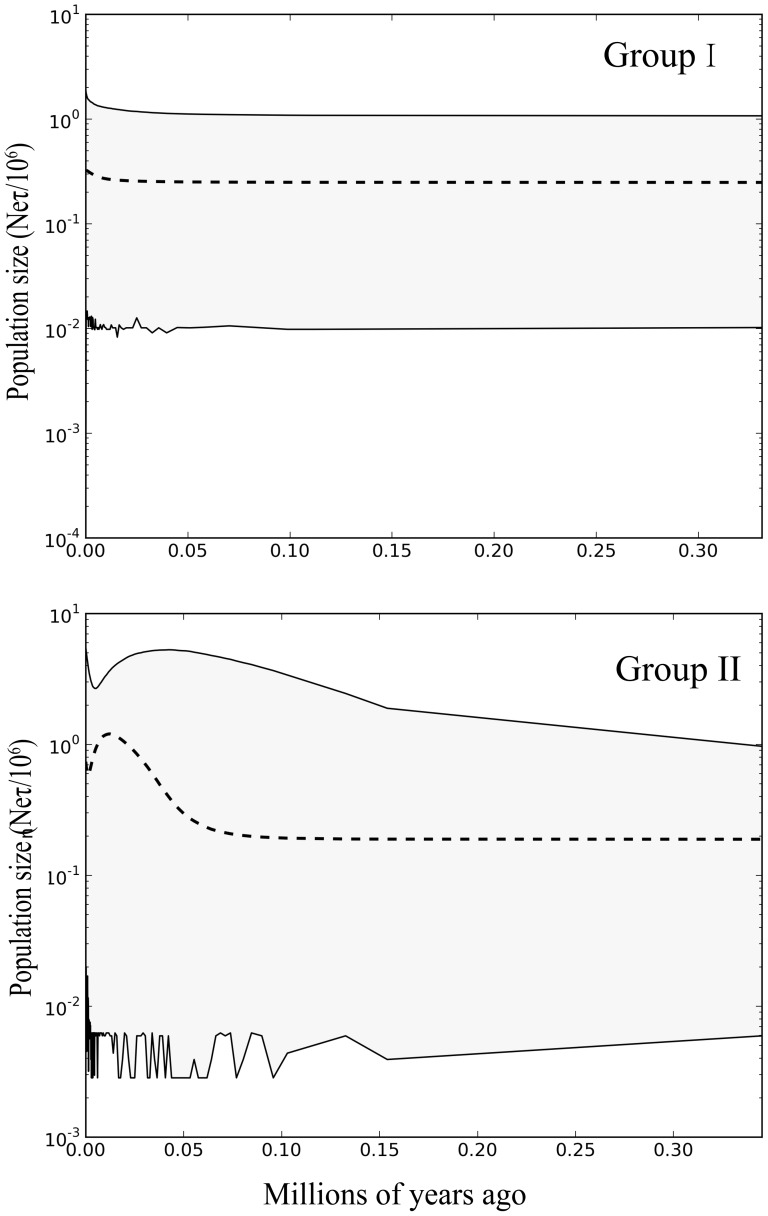
Extended Bayesian skyline plots showing the demographic trends in Groups I and II of *Calotes versicolor*. The x-axis is in units of million years ago and the y-axis represents the estimated population size on a log scale (N_e_τ/10^6^, the product of the female effective population size and generation length in years). The central line shows the median estimate of effective population size, while dashed lines represent the 95% credibility limits.

The mismatch distributions of Groups I and II were multimodal under sudden- and spatial-expansion models, respectively (Figure S2). The sum of squared deviations and Harpending's raggedness index of each group have small and insignificant values (Table 3). Moreover, the values of Fu's *Fs* and Tajima's *D* are significantly negative (Table 3), which rejected the null hypothesis of neutral evolution or population equilibrium. The skyline-plot analysis indicated that the population size of Group I remained relatively stable through time (Figure 3), with the estimated number of changes in population size being effectively zero. In contrast, there was evidence supporting a change in population size in Group II. Based on τ values, the population expansion of Group II under a sudden-expansion model was estimated at about 0.018 Ma. This followed the retreat of the last glacial maximum, as was the case under a spatial-expansion model.

**Table 3:** Mismatch distributions analyses of sudden- and spatial-expansion models and neutrality tests of Groups I and II.

	Parameters	Group I	Group II
Sample size	n	75	137
Sudden expansion model	segregating sites	108	200
	SSD	0.01	0.00
	HRI	0.02	0.00
	τ	3.988 (1.773–9.969)	11.535 (7.246–27.473)
Spatial expansion model	segregating sites	108	137
	SSD	0.01	0.00
	HRI	0.02	0.00
	τ	3.773 (1.863–7.400)	9.406 (6.791–29.019)
Neutrality tests	Tajima's *D*	−2.05**	−1.61**
	Fu's *Fs*	−24.64***	−23.87***

** *P*<0.01;

*** *P*<0.001.

### Ecological divergence

**Figure 4:**
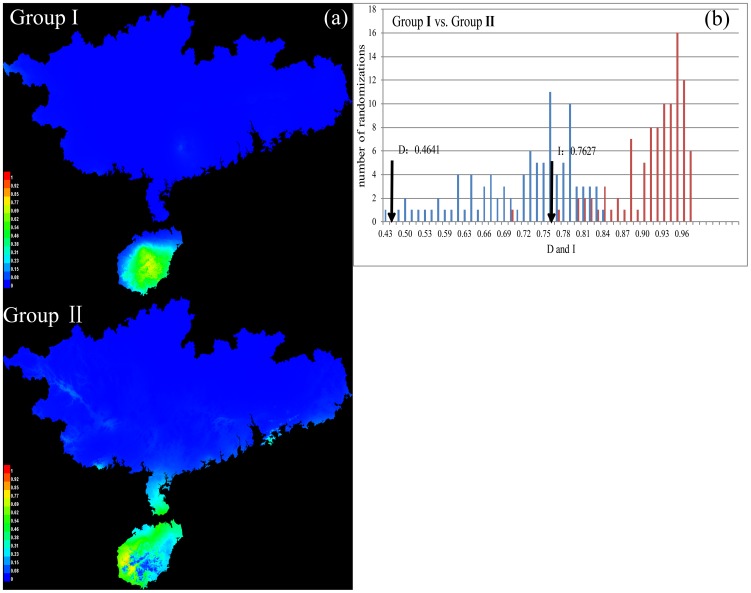
(a) Predicted distributions of Groups I and II generated in Maxent. (b) The results of a niche identity test. Blue columns indicate the null distribution of D, which measures the similarity of two niches. Red columns indicate the null distribution of I, which is an measure of similarity based on the probability distributions of two ecological niche models. The x-axis indicates the value of *D* and *I*, whereas the y-axis shows the number of randomizations. The arrow indicates the value in actual Maxent runs.

The predicted distribution of ecological niche models of the two genetic groups was shown in Figure 4. Areas under the “receiver operated characteristics” curve had values higher than 0.98 in all analyses, indicating that the predicted distribution for each group was robust. Based on our estimates of niche overlap, the observed values of *D* and *I* were significantly different (t-test, *P*<0.001) from the null distributions obtained from the pseudoreplicated data sets.

The principal-components analysis revealed three components that cumulatively explain more than the 89% of the variation, with each principal component (PC) responsible for 42%, 33%, and 14% of the variation, respectively. Precipitation variables dominated PC1 (driest quarter, driest month, coldest quarter, seasonality) and PC2 (wettest month, wettest quarter, precipitation). PC3 loaded mainly on minimum temperature of coldest month as well as mean temperature of driest quarter. The MANOVA analyses indicated that climatic conditions differed significantly between the two groups (Figure S3) (Pillai's trace = 0.58, *P*<0.001). Moreover, there are no significant differences among groups along the first three PC axes (PC1 axis: F_1, 37_ = 0.07, *P* = 0.799; PC2 axis: F_1, 37_ = 1.87, *P* = 0.181; PC3 axis: F_1, 37_ = 1.87, *P* = 0.181).

## Discussion

### Phylogeographical pattern and divergence between major lineages

Our phylogenetic analyses revealed two mitochondrial lineages, A and B, across the sampled populations of *C. versicolor*. The estimated coalescence time for the two lineages is about 0.26 Ma (95% CI: 0.06–0.61 Ma), corresponding to a period of pronounced glacial cycling during the Middle Pleistocene [12], [13]. The inferred coalescence time for lineage A was 0.05 Ma (95% CI: 0.01–0.13 Ma), more recent than that of its sister lineage B (0.13 Ma, 95% CI: 0.02–0.31 Ma), which corresponds to the penultimate interglacial period of the Pleistocene when Qiongzhou Strait was exposed [13], [14]. These estimated coalescence times suggest that *C. versicolor* colonized Hainan Island recently (long after its formation), probably within the Middle Pleistocene when land bridges formed. An alternative possibility is that they reached the islands much earlier but were isolated recently, as a consequence of Pleistocene climatic oscillations and sea-level fluctuations, as appears to be the case for other species in this region [61]–[63].

Our analyses of molecular variance identified two distinct groups, I and II, with restricted geographical distributions. Levels of sequence divergence and frequencies of private haplotypes are high for intraspecific data across such a small geographical area. This pattern suggests that although the populations of *C. versicolor* within our study area share a relatively recent evolutionary history, there has been sufficient time for them to acquire unique haplotypes. However, given that there has been limited gene flow occurring between Groups I and II, despite the absence of any obvious physical barrier, we propose that non-physical barriers have led to geographical subdivision. There are several plausible scenarios for this, as explained below.

One possibility is that species that have small dispersal distances are likely to show distinct genealogical lineages even without geographical barriers to gene flow [64]. Our results suggest that the patterns in *C. versicolor* are consistent with the isolation-by-distance model [65], in which gene flow decreases with increasing geographical distance because of limited dispersal. We have few data on dispersal distance in *C. versicolor*, but it is believed that lizards have limited dispersal abilities [66], [67], which can affect gene flow among populations [68].

The second possibility is that non-physical barriers were stronger in the past. Our ecological niche modeling and niche-identity tests indicate significant niche differentiation between Groups I and II with respect to the selected bioclimatic variables (Figure 4). This result is confirmed by principal-components analysis and MANOVA analysis (Figure S3), which reveal that the two groups are well separated in environmental space. It has been suggested that ‘soft allopatry’ (e.g., ecological disruption) can promote lineage formation across nonphysical barriers, whereby unsuitable habitat reduces dispersal abilities and influences range limits [69]. Adaptation to local environments will yield ecological divergence between populations. This process might play an important role in diversification in *C. versicolor*. Further studies involving additional molecular markers and data on behavior and landscape processes will improve our understanding of the evolutionary history of this species.

### Do Qiongzhou Strait and Wuzhi Mountain impede gene flow between populations?

In the ‘North’ clade, a member of lineage B (see Figure 2), three populations from the mainland (population 1, 2, 3; see Figure 1 and Table 1) form a monophyletic subclade with high support (PP = 0.99). However, the Haian population (population 4) on the mainland falls outside this subclade. Furthermore, in the ‘North clade’, the populations from the mainland and Hainan Island are not reciprocally monophyletic. In addition, the IBD test of the “Qiongzhou Strait isolation” hypothesis suggested that geographical distance contributes to genetic structure between mainland and island populations. These together imply that the neither Qiongzhou Strait nor Gulf of Tonkin acts as barrier to gene flow between populations. However, this interpretation should be regarded cautiously, because similar genetic patterns can result from incomplete lineage sorting. In addition, colonization events or introgression and recent hybridization might have occurred between the island and mainland populations during times of connection.

Unlike those of Reeves's butterfly lizard (*Leiolepis reevesii*), populations of *C. versicolor* on both sides of Wuzhi Mountain did not become reciprocally monophyletic and were embedded within lineage A. This implies that Wuzhi Mountain does not constitute an important barrier to gene flow between *C. versicolor* populations. This was not surprising, considering that the specific habitat requirements of *L. reevesii* are associated with littoral sand and not with the montane conditions in Wuzhi Mountain and high altitudes (>150 m) [63], as for *C. versicolor* which inhabits this mountain ranges [21]. Therefore, Wuzhi Mountain was found to be an important barrier limiting gene exchange for *L. reevesii* [63], but not for *C. versicolor*. Our results suggest that it is necessary to study several species within the same region to see if they share a common phylogeographical pattern, in which case those patterns are likely to have specific geographical causes.

### Demographic history

Our analyses show that Group I has experienced long-term population stability through time, whereas Group II has undergone a moderate population expansion and contraction. The population size and range of Group I was not negatively affected by major Pleistocene climate oscillations, despite changes in sea level affecting the size of Hainan Island. To allow such population stability across the complex environmental gradient throughout alternating periods of glacial and interglacial episodes, intrinsic and extrinsic factors might have limited species dispersal [70], [71]. Despite a lack of apparent physical barriers between groups, ecological factors might have restricted potential movement across unsuitable habitat or prevented dispersal to novel communities. Additionally, interspecific interaction between pre-established and post-differentiated lineages might have played an important role in limiting dispersal abilities and reducing fluctuations in population sizes and ranges. The trans-continental snake (*Diadophis punctatus*) [70], pygmy salamander (*Desmognathus wright*) [71], and spotted salamander (*Ambystoma maculatum*) [72] exhibit a similar pattern. Thus, we propose that habitat requirements and competitive interactions between lineages, rather than the effects of climatic oscillations during the recent glacial periods, have been responsible for the relative population stability in Group I.

The historical demography of Group II seems to be more complex than that of Group I. The skyline plot indicated that the expansion of Group II preceded the last glacial maximum, corroborating studies of other taxa at low latitudes, such as Japanese pipistrelle bat (*Pipistrellus abramus*) [61], intermediate horseshoe bat (*Rhinolophus affinis hainanus*) [62], Reeves's butterfly lizard (*L. reevesii*) [63], and Chinese bamboo partridge (*Bambusicola thoracica thoracica*) [73]. Our estimates are not in line with the expected population contractions and postglacial expansions exhibited by many temperate-adapted species. It appears that habitat preferences and inter-lineage interaction have had a greater effect than the last glacial maximum in shaping the population of Group II.

## Conclusion

We have presented the first comprehensive phylogeographical analysis of the Oriental garden lizard (*Calotes versicolor*) on Hainan Island and the adjacent mainland. We identified two mitochondrial lineages occupying specific habitats separated by putative nonphysical or ecological barriers. Although our study is based on a single locus (mitochondrial DNA), we have been able to gain some useful insights into the demographic history of *C. versicolor*. We proposed that Pleistocene sea-level fluctuations had profound effects on lineage diversification and regional genetic structure. However, the demographic history of the species was influenced by habitat requirements and competitive interactions between lineages, rather than changes in climate during the last glacial maximum. Our study highlights the importance of nonphysical barriers, such as ecological processes and geographical distance, in shaping the present-day distribution of a species at lower latitudes in tropical China. We suggest that the addition of ecological niche modeling to phylogeographical analyses will offer further insights into the mechanisms that determine the distributions of other organisms in this region.

## Supporting Information

Figure S1 **The complete time-calibrated** ***Calotes versicolor*** **phylogeny used for secondary calibrations.** Branch lengths are in units of time. Node bars indicate 95% credible intervals of estimated divergence times. Red bars indicate fossil-calibrated nodes. Posterior probabilities above 0.50 are shown next to nodes.
(TIF)

Figure S2 **Observed and expected mismatch distributions showing the frequencies of pairwise differences of Group I and Group II based on models of sudden expansion (a, b) and spatial expansion (c, d).**
(TIF)

Figure S3 **Principal-components analysis of climatic niche differences between Groups I and II (framed by circle and square, respectively).** (a) X-axis indicates PC1, and Y-axis indicates PC2. (b) X-axis indicates PC2, and Y-axis indicates PC4.
(TIF)

Table S1 **Accession numbers for the outgroup sequences retrieved from GenBank and used for phylogenetic analyses of Fiure S1.**
(DOC)

Table S2 **Results of the principal components analysis of 19 bioclimatic variables.**
(DOC)
