# Supplementary material for: Diversification and Demography of the Oriental Garden Lizard (Calotes versicolor) on Hainan Island and the Adjacent Mainland
Source: PLoS One. 2013 Jun 26;8(6):e64754. doi: 10.1371/journal.pone.0064754 (PMC3694074; doi:10.1371/journal.pone.0064754)
Supplement: Table S1 — Accession numbers for the outgroup sequences retrieved from GenBank and used for phylogenetic analyses of Fiure S1. (DOC) [file pone.0064754.s004.doc]

**Supplementary Table S1**

**Table S1**. **Accession numbers for the outgroup sequences retrieved from GenBank and used for phylogenetic analyses of Fiure S1.**

| Taxon | Accession number |
| --- | --- |
| Iguanidae |  |
| *Iguana iguana* | NC_002793 |
| Chamaeleonidae |  |
| *Chamaeleo africanus* | EF222197 |
| *Chamaeleo dilepis* | EF222189 |
| Leiolepidinae |  |
| *Leiolepis belliana* | U82689 |
| Amphibolurinae |  |
| *Physignathus cocincinus* | PCU82690 |
| *Pogona vitticepes* | AB166795 |
| Hydrosaurinae |  |
| *Hydrosaurus amboinensis* | NC_014178 |
| Agaminae |  |
| *Agama agama* | AF128504 |
| *Agama bibronii* | AF128506 |
| *Laudakia caucasia* | AF028683 |
| *Laudakia microlepis* | AF028678 |
| Draconinae |  |
| *Acanthosaura armata* | AB266452 |
| *Calotes emma* | DQ289460 |
| *Calotes liolepis* | AF128485 |
| *Calotes irawadi* | DQ289468 |
| *"Calotes versicolor"* | DQ289469 |
| *"Calotes versicolor"* | DQ289470 |
| *"Calotes versicolor"* | DQ289471 |
| *"Calotes versicolor"* | DQ289472 |
| *"Calotes versicolor"* | DQ289473 |
| *"Calotes versicolor"* | DQ289475 |
| *"Calotes versicolor"* | DQ289476 |
| *"Calotes versicolor"* | DQ289477 |
| *"Calotes versicolor"* | DQ289478 |
| *Draco blanfordii* | AF128477 |
