# Supplementary material for: Diversification and Demography of the Oriental Garden Lizard (Calotes versicolor) on Hainan Island and the Adjacent Mainland
Source: PLoS One. 2013 Jun 26;8(6):e64754. doi: 10.1371/journal.pone.0064754 (PMC3694074; doi:10.1371/journal.pone.0064754)
Supplement: Table S2 — Results of the principal components analysis of 19 bioclimatic variables. (DOC) [file pone.0064754.s005.doc]

**Supplementary Table S2**

**Table S2** **Results of the principal components analysis of 19 bioclimatic variables.**

|  | Name of bioclimate data | PC1 | PC2 | PC3 |
| --- | --- | --- | --- | --- |
| bio1 | Annual Mean Temperature | .096 | -.522 | .832 |
| bio2 | Mean Diurnal Range: Mean of monthly (max temp - min temp) | -.299 | .005 | -.127 |
| bio3 | Isothermality: (P2/P7)* 100 | -.605 | .225 | .438 |
| bio4 | Temperature Seasonality (standard deviation *100) | .517 | -.287 | -.759 |
| bio5 | Max Temperature of Warmest Month | .594 | -.736 | .223 |
| bio6 | Min Temperature of Coldest Month | -.013 | -.256 | .954 |
| bio7 | Temperature Annual Range (P5-P6) | .442 | -.273 | -.801 |
| bio8 | Mean Temperature of Wettest Quarter | .596 | -.612 | -.159 |
| bio9 | Mean Temperature of Driest Quarter | -.247 | -.221 | .907 |
| bio10 | Mean Temperature of Warmest Quarter | .452 | -.764 | .313 |
| bio11 | Mean Temperature of Coldest Quarter | -.237 | -.193 | .945 |
| bio12 | Annual Precipitation | .326 | .931 | -.099 |
| bio13 | Precipitation of Wettest Month | .050 | .967 | -.029 |
| bio14 | Precipitation of Driest Month | .941 | .151 | -.209 |
| bio15 | Precipitation Seasonality (Coefficient of Variation) | -.854 | .204 | .127 |
| bio16 | Precipitation of Wettest Quarter | -.044 | .955 | -.087 |
| bio17 | Precipitation of Driest Quarter | .944 | .131 | -.196 |
| bio18 | Precipitation of Warmest Quarter | .400 | .736 | -.447 |
| bio19 | Precipitation of Coldest Quarter | .923 | .151 | -.281 |
